# Supplementary material for: Patterns of Intron Gain and Loss in Fungi
Source: PLoS Biol. 2004 Nov 30;2(12):e422. doi: 10.1371/journal.pbio.0020422 (PMC532390; doi:10.1371/journal.pbio.0020422)
Supplement: Table S1 — Also available at http://genes.mit.edu/NielsenEtAl/. (4.3 MB ZIP). [file pbio.0020422.st001.zip › NielsenEtAl/html/1325.html]

AN2751.1.NCU06540.1.MG08693.1.FG09663.1


```
 CLUSTAL W (1.82) Multiple Sequence Alignments - Introns Inserted


Sequence 1: MG08693.1	826 aa
Sequence 2: FG09663.1	812 aa
Sequence 3: NCU06540.1	827 aa
Sequence 4: AN2751.1	819 aa
Alignment Length: 877 aa
Number Identitical Residues: 298 aa
Alignment Score (without introns) 14895


MG08693.1 	MSFFGFDTSGHNKGAP-----GFSQAHDPFAGL-------GQDSSGEA~LDFDDTYDGLG
NCU06540.1	MSFFNFDQSRHNTAAP-----GFSQAHDPFASLA------NRDEEAEE~IDFEDTYDGLG
FG09663.1 	MSFFGFDNARHNTAAP-----GFSQAHDPFAGLS------GKDGDDDA~LDFEETYDGLG
AN2751.1  	MSFFGFDTTLPRDRAPQGGQRGIFDTPDPFAEVARARAAAQHEDDDDV2LNFEDTYDGLG
          	****.** :  .  **.... *: :: **** :: : ::: :: . :  ::*::******

MG08693.1 	DQLDETDDAFNDDTFG-GGDSTDKKVGKDFDFFGQTAKVANAIDEEHMRYNRSQPTQRPA
NCU06540.1	DQLDETDDAFNDDTFG-GDTGEPGPVGKDFDFFGHTAKVANVIEEENVRFNRQQPAAKAP
FG09663.1 	DQLEETGDAFNDDTFGDSGPAVSRNAGNDFDFFGQTAKVADAIEEEHVRFHRQKPTAKLA
AN2751.1  	DQLDDDQDAFNEDTFG---DVGSGPVGKDFDFFGKTAQVSDVIGEEQVRYNLKNPQASVA
          	***::  ****:****         .*:******:**:*::.* **::*:: .:*    .

MG08693.1 	QPIP--------EYQ--YS----MQQQSAPRPVRSGYEKYKESEPVPDMQVDAALWGVAP
NCU06540.1	QPVAQP-----SAYTPIAGPSYNYFAQAPVRPARTGYEKYHEQ-PIEDLQVDAALWGVAP
FG09663.1 	QAPPPVDQYAPTDYQSYYSGQPYQQQPFQQQPVRSGYEKYREPEPLPDLHVDRSIWGIGP
AN2751.1  	PRATEP---------------QKTATTTAQQPKRTGYEKYSDPDYIPDLQAKSSVWGLPS
          	   .                          :* *:***** : . : *::.. ::**: .

MG08693.1 	KKQASQA-PAPIPVAS-----ASRKMMSLEEVEASMRAQSSQPSEFAAPGSYPGHGQLAH
NCU06540.1	KKQPPPPAQPAVPSAPS----GGRKMLSLEEVEAQMRAQAKAATPSPAPQHQPHQIPHEQ
FG09663.1 	SKPAQQASPAPVPAAQPVQHGASRKVMSLEEVEAAMRSQPMQHT------PQPHAADLSY
AN2751.1  	K-------PESAPQATT----QTKKMMSLEEVEAQIRNQGAAAPGFGPQVSLPQHVPEPP
          	.         . * * .      :*::******* :* *    .   .    *       

MG08693.1 	QGFLPQQGADYLYAQQQQVRHVPSPTAHLQHGQGQPSLAILQRPQSRSPADSMPHGLPQG
NCU06540.1	AYLYQHQAQNQPQAHFQAG----VVEPLYHQPQGHP-MTILQRPHSKPPTPAQPS--SQP
FG09663.1 	Q----QPPPGFAGHQHQ---------PPQAHGHGHP-VAILQRPQSTQSKSTPPAPGLQG
AN2751.1  	LP--LQRPPGLPEGFPQLP----PEYLEAQFGRGVP--PQFLHPRTIAPEPFPHPAQAPN
          	     :   .      *               :* *  . : :*::  .           

MG08693.1 	LAPQQQRHAQAHRQPSPQGPSTKPMQILQNPNRHPGDNVRLGS----------QPNHQGH
NCU06540.1	PAIQQHQQPAVPQQ---------PTQILQNPNRLSGDAAKIGLPVHPTPPSGPQSAHQGH
FG09663.1 	TPVQHHQQPHAN-----------PTQILQNPTRT-GSDAHAPH----------QQGQGAK
AN2751.1  	IPLHLLQNPIQNPN-------APPSHMGPLQHQAIPSRDQPPQ----------RLPQQHL
          	 . :  ::.    .       : * ::     :   .  :             :  :   

MG08693.1 	RQQN-SFS-QTGILTHPSQLSQLSDEEKNAYLEAEARRAKRNHKIFMLSKDNGLMTPQDK
NCU06540.1	RQQG-SLPRQPPMILHPAQLAQLSEEEKAAYLEQEAKRAKRNHKIFQMSRDNGIMTPQDK
FG09663.1 	QSHG-AVP-SAAQLQAHPQMQQMSEEEKAAFLDQETKRAKRNHKIWLLSKDNGLMTPQDK
AN2751.1  	PQHGRGLSAGLPLITNPQQLMNLTEEQRVAYLMEDAKRAKRNHKIFLLSKGNGLMTPQDK
          	 .:. ...     :    *: ::::*:: *:*  :::********: :*:.**:******

MG08693.1 	NFITRIQLQQLVAATGNPMDQGADDHIAEDFYYQVHSQIRGGHRQHPGQPLNNFAQTYLY
NCU06540.1	NFITRIQLQQLVAATGNPNEHGTDESLAEDFYYQVHSQIQAGHRQHPSQPLNQFAQTYLF
FG09663.1 	NFITRIQLQQLVSATGNPVE-GSDASIAEDFYYQVFSHIRAGQRQNPSQPLSNFAQTYLY
AN2751.1  	NFITRIQLQQLVAQAGNMADTDSDAVLAEDFYYQVYSQIRGAPRQHPHQPLGHFAQTYLL
          	************: :**  : .:*  :********.*:*:.. **:* ***.:****** 

MG08693.1 	QTGSRQAG-ARRHNRGPENHVQRMEQQVQRAVEAAKSKPKNKQLVIEGSLGKISFSNAKT
NCU06540.1	QTGSRQGG-MRRHNRGPENHMQRMEQQVQRAVEAAKNKPKNKQLVIEGSLGKISFSNAKT
FG09663.1 	QTGSRQGG-MRRHGRPAENHFQRMEQQVQRAVEAAKNKPKNPQLVIAGSLGKISFSNAKT
AN2751.1  	QTGNRIGGHSRKHGQSADNHMQRMQQQVQRAVEAAKAKPKNKQLIIEGSLGKISFGNAKA
          	***.* .*  *:*.: .:**.***:*********** **** **:* ********.***:

MG08693.1 	PKPLLNIKRTDSASDANRPGSSHKAPSHSAIINRKTVLSDIEKVYETLMRMEDHDRKMPP
NCU06540.1	PKPLLNIKRNDSASDVKKAPHPVNGRD------KKDELRALELVYTTLMKMEDHERNMPM
FG09663.1 	PKPLLSIKRTES--EHQRPNNGKKGSS----LDRKTILRNVEKVYQTLMQIEDHVRLIPP
AN2751.1  	PKPSLNIKRPESSEGGKATKKLPTDLSP---SDRKSILANLEGVYNTLMELEDMARTMPP
          	*** *.*** :*:.  : .    .  .     .:*  *  :* ** ***.:**  * :* 

MG08693.1 	PNDSSDPELVRVQMEWQQGAAQLNNQLWTNLKVHEPIGAT--AIHPFIAFLSYPKGKKAL
NCU06540.1	APAEDDQEALAKVFAWSNEGQALNEQLWKALRVQDHDQPG--RIHPFMALLSYSKGKKAI
FG09663.1 	PMTSPDEELENKHKEWAIILESYNAKLWEELKVHEQIGVV--VPHPFIAFLSCAKGEKAI
AN2751.1  	PPDENDSEAIQTHMEWRQKLNALNQKLWNQMKVMEAIVPNSNTPHPFIALLSYPKGKKAI
          	.  . * *       *       * :**  ::* :     :.  ***:*:** .**:**:

MG08693.1 	PRVFRHISYEQRCTILTMIVVHLDKIDVVRDAATDAGET-LLNAALRENIELFSVAVMST
NCU06540.1	PRIFRHTNHEQRITIFTRIVVNLDQLDVVREAQVTTDDI-QFNAAMRENIELFSAAIVTT
FG09663.1 	PRLFPHLSFEQRTTILTMIIYHLDQLDVVQGAAITPGEVTTINARMREKIELFISTVMPS
AN2751.1  	PRIFRHIDQEQRITILTMIVVNLDNLDVVRRALPAPGEA-QPPLAAREAIDLFSQAVMPC
          	**:* * . *** **:* *: :**::***: *   ..:        ** *:**  :::. 

MG08693.1 	LFNFMNELNLDIVTGVLGIISG-LSVDTIARTRIGASMLTMILSRAEILKQGGA-PQQLI
NCU06540.1	LFTFMNELGLDLVAAILGLVCT-RNVDVIAKSRIGASMLTMILSRAELIKHNGGGSEQDW
FG09663.1 	LMQYFNDTGLDIVDGVLNLIATKLNVDLIARSRIGVSMLTLILSRAVLLKQTGAGTPEQW
AN2751.1  	LLGYVNEAPFNIIIGLLGLVLTHTHAQFVARTRIGLGILTMLLSRAEIVKEAGQASEQDW
          	*: :.*:  :::: .:*.::     .: :*::*** .:**::**** ::*. * .. :  

MG08693.1 	EQW2NNTYNNFFTLLEPTLPRIFPGSVNSGQDIYVWQLLAALGIGANQDQQQRLVLGVK~
NCU06540.1	RSW2DMTYTQFFNILEPTLPYIFPGSIATDQDTYVWQLLAAIGIGASPDQQQRLVIAVK~
FG09663.1 	DNW2DRTFEILFTRLEPSLPYIFPGSVNTGEDVYVWQLLAAMGVSATHDQQTRLVLAVK~
AN2751.1  	KQW~VEKFNILFDTLEPTFADIFPNSINAGDDMYVWQFLAAIGIGASPEQQQRLVIAVK2
          	 .*   .:  :*  ***::. ***.*: :.:* ****:***:*:.*. :** ***:.** 

MG08693.1 	DRVMDTVALSKTLPQDMAQARLQNVNLFMRSIGLDVELLV
NCU06540.1	DRVMDTVSLAKTLPPALAADRLQNVNLFMRSIGLDVELLQ
FG09663.1 	DRVLDTVTVSKTLPPVMGAERLNSVNLFMRSIGLDVELLQ
AN2751.1  	DRVMETVTHSKTLPADMASQRLGNVNLFMRAIGLDVELLG
          	***::**: :****  :.  ** .******:********
```
